# Research participants’ perceptions and views on consent for biobank research: a review of empirical data and ethical analysis

**DOI:** 10.1186/s12910-015-0053-5

**Published:** 2015-09-09

**Authors:** Flavio D’Abramo, Jan Schildmann, Jochen Vollmann

**Affiliations:** 1 Institute for Medical Ethics and History of Medicine, Ruhr-Universität Bochum, Markstraße 258a, D-44799 Bochum, Germany; 2 Charité Comprehensive Cancer Center, Charité - Universitätsmedizin, Charitéplatz 1, Berlin, D-10117 Germany

**Keywords:** Consent, Research ethics, Genomic research, Biobank

## Abstract

**Background:**

Appropriate information and consent has been one of the most intensely discussed topics within the context of biobank research. In parallel to the normative debate, many socio-empirical studies have been conducted to gather experiences, preferences and views of patients, healthy research participants and further stakeholders. However, there is scarcity of literature which connects the normative debate about justifications for different consent models with findings gained in empirical research. In this paper we discuss findings of a limited review of socio-empirical research on patients’ and healthy research participants’ experiences and views regarding consent to biobank research in light of ethical principles for appropriate information and consent.

**Methods:**

Review question: Which empirical data are available on research participants’ perceptions and views regarding information and elicitation of consent for biobank research? Search of articles published till March 1st 2014 in Pubmed. Review of abstracts and potentially relevant full text articles by two authors independently. As categories for content analysis we defined (i) understanding or recall of information, (ii) preferences regarding information or consent, and (iii) research participants’ concerns.

**Results:**

The search in Pubmed yielded 337 abstracts of which 10 articles were included in this study. Approaches to information and consent varied considerably across the selected studies. The majority of research participants opted for some version of limited consent when being informed about such possibility. Among the factors influencing the type of preferred consent were information about sponsoring of biobank research by pharmaceutical industry and participants’ trade-off between privacy and perceived utility. Studies investigating research participants’ understanding and recall regarding the consent procedure indicated considerable lack of both aspects. Research participants’ perceptions of benefits and harms differ across those studies.

**Conclusion:**

The knowledge, perceptions and views of research participants who have undergone a consent procedure within the context of biobank research raise several questions on the issue of how to inform and elicit consent in an ethically acceptable way. In our empirical-ethical analysis we develop suggestions on how the practice of eliciting consent in the biobank context should be improved.

## Background

The achievements of the Human Genome Project and the use of technologies enabling researchers to analyze large amounts of DNA have made biobanks an established part of research practice [1]. Biobanks contribute to a multitude of ongoing research projects by storing huge amounts of biological information and data about phenotypes, clinical aspects and also, in some cases, lifestyle information, such as nutrition and exercise. One of the earliest and most intensely discussed questions from an ethical as well as legal perspective with regards to biobank research is that of appropriate consent to biobank research. The fact that the nature of the research and its risks and benefits to potential participants often cannot be disclosed in detail at the time of eliciting consent has been seen by some scholars as a challenge to “informed consent” [2–4]. According to the standard account of informed consent, individuals are considered as sovereign with regards to decision-making about themselves [5]. The demand for informed consent is not a movement stemming from within medical research or practice, but can be better described as a development pushed into medical practice in the form of ethico-legal regulations—also as a reaction to various scandals in medicine [6, 7]. In addition and connected with the birth of bioethics in the 1960s, informed consent has been emphasized as an antidote to paternalistic approaches in medicine [5, 8].

**Fig. 1:**
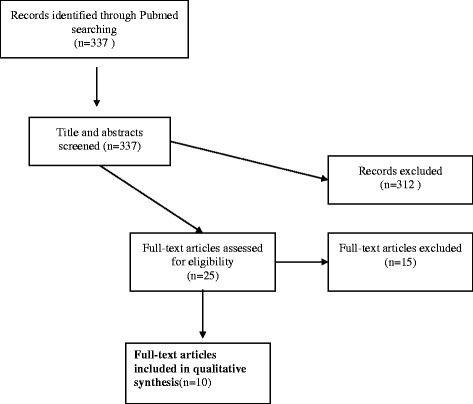
Flow chart literature search

The development and justification of alternative consent models has been at the center of interdisciplinary research [9–12]. On the one hand, it has been argued that the use of informed consent as it was formerly conceived is difficult to realize with the rise of new technologies related to big data and their application in medicine (i.e. the protection of individual privacy stands in contradiction to the gathering of data collected in big databases). On the other hand, the roles of individuals is more and more emphasized, for example, in the form of regulations to protect individual rights (i.e. privacy), with the result that scientific practices, such as population registries, are challenged because of new laws emphasizing the use of informed consent for scientific practices which are of benefit for local communities [13]. This tension described previously has resulted in the development of a strategic planning reform of science introduced by the European Commission, called “Science 2.0,” or “Science in Transition” [14], strategic planning also extended to biobanking [15], which can be characterized as approaches to include the voices of all the stakeholders (i.e. patients, citizens and researchers).

While an analysis of the multitude of models of consent developed and their philosophical, ethical and legal foundation are beyond the scope of this paper, one way of structuring the debate is by distinguishing the forwarded models as “narrow” versus “broad” consent to biobank research. According to this distinction, the narrow consent model shares key assumptions of informed consent, in the sense that consent to biobank research should be specific and based on concrete information about the research planned [16]. As one consequence, participants who give consent to a defined project need to be recontacted if the biological material and/or data is intended for use in another research project. Narrow (informed) consent has been criticized for not taking the practical challenges sufficiently into account, such as acquiring consent from dead donors, drop-out rates that decrease the scientific value of the study or substantial logistic expenses [17–19]. In broad consent, patients or research participants give their consent to the use of biological and/or clinical data in research which is not specified at the time of consent. There are different lines of argument in favor of such broad consent. One line is that the risks of biobank research are perceived to be small compared to the related benefits [20]. This risk assessment is sometimes combined with a second argument that holds a duty of solidarity to support medical research with such a risk-benefit ratio [21–23]. Thirdly, broad consent has been supported for pragmatic reasons, as it appears to be the more efficient regarding the economic and logistic aspects of biobank research [24].

Narrow and broad models of consent differ in ethically relevant aspects, mostly because a broad model of consent confers more responsibilities on institutional bodies (e.g. ethics committees and institutional review boards) and justifications forwarded for limitations of information given to the individual research participant on research aims, risks and benefits [25]. By contrast, narrow models of consent stress much more the role of participants’ understanding of details of the research and their individual rights with regards to the conduct of research. Nevertheless, broad and narrow models of consent also share assumptions. One example is that in order to give valid consent, participants should be enabled to understand the issues at stake [26]. In the wake of the strategic planning of science and biobanking [14, 15], many socio-empirical studies have been conducted during recent years to gather the experiences, preferences and views of patients, healthy research participants and further stakeholders with regard to consent in the context of biobank research. However, there is scarcity of literature which connects the normative debate about justifications for different consent models with findings gained in socio-empirical research. Against this background, we summarize the findings of a limited review of socio-empirical research on patients’ and healthy research participants’ experiences and views regarding consent to biobank research. We focus in our subsequent empirical-ethical analysis on findings which, in our view, are informative to answer the question of what an ethically acceptable practice of informing potential research participants and eliciting consent should look like.

## Methods

In a first step, we formulated the review question: Which empirical data are available on research participants’ perceptions and views regarding information and elicitation of consent for biobank research? For the purpose of this review biobank was defined as structure in which human biological and/or clinical data is stored for the purpose of research. As the second step, we chose PubMed as database to query. This decision was made in light of the fact that our past work on the topic as well as the conduct of systematic reviews on other ethical topics indicates that use of this database generates the vast majority of relevant articles [27–29]. In a third step we developed a search algorithm based on key words in relevant conceptual and empirical studies: (consent [tw] OR privacy [tw] OR autonomy [tw]) AND (biobank [tw] OR genomic research [tw] OR personalised medicine [tw] OR genomic data [tw]. In a fourth step, criteria to determine the relevance of retrieved articles were defined. These criteria are based on the review questions as well as a sample of studies about the topic known to the researchers prior to the review [5, 18, 30–34]. Table 1 summarises in- and exclusion criteria.

**Table 1:** Inclusion and exclusion criteria

Inclusion criteria	Exclusion criteria
Research method/Type of publication	
Empirical research (qualitative or quantitative)	Case studies
	Theoretical papers
	Comments or opinion papers
	Guidelines
Research participants	
Research participants have been involved in consent procedure for biobank research	Empirical research on perception or views of public or patients not involved in biobank research
	Empirical research focusing paediatric genetic research (i.e. view of parents/guardian).
Topic of research	
Research participants’ perception or views on consent is central topic of the study	Studies on biobank research participants’ views or attitudes other than consent
	Papers focusing on disclosure of incidental findings

The search was limited to articles published till March 1st 2014. All titles and abstracts were read by two authors (FD, JS) and a decision regarding the inclusion or exclusion of the articles based on the predefined selection criteria was made independently. Where no abstract was available, articles with a potentially relevant content (as indicated by title) were ordered and the decision was made after reading the full text. In case of disagreement between both authors consensus was sought based on the abstract or if necessary full text was retrieved (Fig. 1).

### Content analysis

The following categories were defined prior to data analysis with the aim of quantification of data: (i) nationality of research participants, (ii) target groups (i.e. healthy research participants, patients as research participants), (iii) research methods (i.e. surveys, semi-structured interviews, focus groups and other forms of group discussion). In addition and for the purpose of qualitative content analysis which follows basic principles as described by Mayring [35], the following pre-defined categories were formed: (i) understanding or recall of information, and (ii) preferences regarding information or consent. Both categories reflect the interest of the researchers in light of their preconceived knowledge from the literature prior to the research. The first category was thought as pivotal in the process of giving an informed consent as comprehending the information received is the very base on which taking an autonomous decision and because the issue of understanding is one of the weakest points of informed consent [5]. The second category was ideated as it gives further indications useful in making an informed consent process based on preferences of research participants. In the course of the systematic literature we added the category (iii) research participants’ concerns. This third category was developed in a bottom up way based on a first analysis of relevant articles in which we identified expression of concerns from biobank’s participants as a topic relevant for the context of our review.

## Results

The search in PubMed yielded 337 abstracts. Twenty five full text papers were read and assessed against inclusion and exclusion criteria. Of these, finally 10 articles were included in this study [36–45]. Reasons for exclusion at this stage were mainly due to lack of focus on participants’ perceptions or views regarding information or consent, for example [Hobbs et al. [46], Ridgeway et al. [47], Steinsbekk et al. [48], Williams et al. [49], or as the focus was on subjects who had not been involved in a consent procedure regarding biobank research, for example Rahm et al. [50], or because the focus was on consent with parents of children donors, for example Tindana et al. [51].

### Characteristic of studies and research participants

Five studies had been conducted in the U.S. [39–43]. Four of these studies had been conducted by researcher(s) of the Center for Medical Ethics and Health Policy, Baylor College of Medicine, Houston, Texas [39–41, 43]. The other studies had been conducted in Australia [38], Italy [36], Japan [45], Spain, including a small sample from US, [44], and Sweden [37]. Most of the studies had been conducted with patients who were participating in biobank research (*N* = 9) [36, 38–45]. In one of these studies research participants were exclusively patients with cancer [36]. In some studies parents of pediatric patients were involved in addition [Lipworth et al. [38], McGuire et al. [40], Oliver et al. [41], Robinson et al. [43]. The Swedish study presents findings from a random sample of a biobank without further information about the health status [37]. Data collection was performed in seven studies via structured questionnaires which in some cases included open ended questions [36, 37, 40, 41, 43–45]. The study by [Watanabe et al. [45] in addition to quantitative data reports also qualitative findings but without any account of systematic gathering and analysis of the latter kind of data. Ormond et al. [42] use a modified version of the structured Quality of Informed Consent (QuIC) questionnaire [52] combined with a qualitative section of 12 questions on consent. Two studies used qualitative methods [38, 39] for data gathering and analysis. Table 2 summarizes main characteristics and findings of eligible studies.

**Table 2:** Summary of characteristics and findings of eligible studies

Authors / Year of Publication	Country	Research participants	Method of data collection	Main findings
1. Robinson et al. 2014 [43]	USA	229 patients; parents/guardian of paediatric patients and family members as controls	Structured interview and open ended questions	Over 25 % of the research participants did not remember that they signed a informed consent document to participate in a genomic study. The majority (54 %) could not correctly identify with whom they had agreed to share their genomic data. Participants felt that they understood enough to make an informed decision.
2. Cervo et al. 2013 [36]	Italy	430 cancer patients	Structured questionnaire	36.5 % of research participants indicated that they knew what a biobank was before participating in the study. After the multisource informed consent procedure more than 95 % of patients were aware of participating in a biobank project subsequent to the consent procedure. The final assessment showed more than 95 % correct answers. The information received was judged to be “very understandable” by 44.9 % and “fairly understandable” by 53.8 % of the research participants.
3. Oliver et al. 2012 [41]	USA	229 patients; parents/guardian of paediatric patients and family members as controls	Structured interview and open ended questions	Subsequent to information about three different types of consent with different scopes for data sharing, research participants in all three groups indicated that they would be more restrictive compared with their actual decision before debriefing. Qualitative findings support the more skeptical view on data sharing subsequent to being informed about different options of consent with limits to data sharing. Research participants’ trade-off regarding privacy and utility of research was significantly associated with participants’ decision about data release subsequent to debriefing.
4. McGuire et al. 2011 [40]	USA	336 participants, parents/guardian of paediatric patients and family members as controls made initial data release decision prior to be informed about different consent options. 323 research participants made final data release decisions subsequent to debriefing.	Structured interview on data release decision and socio-demographic data.	Before being informed about different consent options 83.9 % of participants chose public data release. After debriefing, 53.1 % chose public data release, 33.1 % chose restricted (controlled access database) release, and 13.7 % opted out of data sharing. Hispanic background, not being married and college degree were associated with choice for restricted data release.
5. Watanabe et al. 2011 [45]	Japan	1378 patients	Structured questionnaire (in addition report of qualitative narratives by research coordinators on patients’ perception)	Information about biobank research was judged as understandable in 76.8 % subsequent to personal discussion compared to 60 % after information via brochures and 56.9 % via DVD. Interest in personal data was positively associated with better recall rate, in younger patients, higher satisfaction with consent process and with willingness to participate in future study. Research coordinators indicate need among research participants to get more information following consent procedure.
6. Valle-Mansilla 2010 [44]	Spain (USA)	279 patients (30 patients from US sample)	Structured questionnaire	230 patients (82.4 %) remembered giving consent to biobank research. 40.3 % preferred general consent for future use of sample, 49.8 % preferred limited or specified consent. Support for broad consent was less supported in industry-sponsored research.
7. Lipworth et al. 2009 [38]	Australia	12 patients, parents of patients and other lay stakeholders	Semi-structured in depth interviews	Participants’ supported making use of material/data subsequent to participation in biobank research. There was evidence for expectation regarding direct benefit of research for research participants and little awareness of harm.
8. Ormond et al. 2009 [42]	USA	200 patients	Structured interviews including open ended question	The best understood domains included the nature of the study, benefit to future patients, and the voluntary nature of participation. Lower knowledge scores included potential risks and discomforts, experimental nature of the research, procedures in the event of study-related injury, and confidentiality issues. Only 10 % of participants explicitly stated they had no expectations for personal benefit.
9. McGuire et al. 2008 [39]	USA	15 patients and controls from a genomic study on epilepsy	Focus groups including one Follow up focus group with presentation and discussion of findings from initial focus groups.	There was a general interest in receiving information and making decisions about data sharing. Participants preferred multiple data sharing options. However, they were more likely to consent to public data release when given fewer options. Most participants felt that genomic information should not be publicly released without explicit consent from research participants.
10. Hoyer et al. 2005 [37]	Sweden	930 research participants	Structured questionnaire	64.5 % of participants were aware that they had consented to donate a blood sample, 55.4 % thought that they had consented to donate phenotypic information, and 31.6 % believed that they could withdraw their consent. 85.9 % acceped surrogate decision making by regional research ethics committees.

### Preferences regarding different types of consent

Five studies reported findings on preferences of research participants regarding the mode of consent to biobank research. Being asked about general preferences for broader or narrower versions of consent, Valle-Mansilla et al. [44] report that the majority (59.8 %) of the 279 research participants preferred some type of limitation to consent. In this study 29.7 % preferred to be re-contacted in case of any new research using the donated material and data. In contrast, Hoeyer et al. [37] report that 85.9 % of the research participants accepted surrogate decision making by regional research ethics committees with regards the use of the blood donated to the biobank. Three studies explored preferences regarding consent with a focus on the choice between different options of data sharing [39–41]. In two of these studies [40, 41] research participants willing to donate biological material into one of six genome studies at Baylor College of Medicine in Houston, randomly underwent three different models of consent which allowed different degrees of choices regarding control of public data release and data sharing. The three models of consent consisted either of giving consent to public data release without alternative (i), a choice between consent to public data release and no release (ii), or a choice between consent to public data release, restricted release only or no release at all (iii). In a follow up visit research participants were informed about all options of data sharing. Following this information 30.8 % less participants than initially consented to public data release (83.9 % versus 53.1 %), 33.1 % chose restricted data release and 13.7 % did not consent to data sharing [40]. The analysis of data on attitudes to consent of research participants following the disclosure of three different consent options has been published in a separate paper by Oliver et al. [41]. This study indicates that research participants’ attitudes to data sharing are even more restrictive after they had been informed about different options regarding data sharing than their actual choice and this had been documented in the study by McGuire et al. [40]. The quantitative findings are corroborated by a qualitative focus group study by McGuire et al. [39]. Here, participants indicated a more restrictive approach towards public data sharing if they received information about different options for control of data. This study shows also that most of the participants who had provided material and clinical data did not support data sharing without explicit consent.

Factors possibly influencing research participants preferences on different types of consent have been explored in the study of Valle-Mansilla et al. [44]. This study indicates that the information about sponsoring of biobank research by pharmaceutical industry was associated negatively with a preference for broad consent. In addition, Oliver et al. [41] show that decisions about the degree of sharing of data were statistically significant associated with research participants’ trade-off between privacy and perceived utility of data sharing. In their study on views of Swedish biobank’s research participants, Hoeyer et al. [37] report that positive experiences with healthcare services and satisfaction with information about the biobank were associated with acceptance of surrogate decision making about the use of biological data by research ethics committees.

### Recall and understanding

Five studies investigated patients’ recall or understanding following information and consent [36, 37, 42, 43, 45]. A general finding of these studies was lack of recall and understanding with regards to the given consent in differing proportions of the research participants. Using data generated in study with an experimental design of three different consent options randomly used for a U.S. biobank’s recruitment of research participants among patients (see above McGuire et al. [40], Oliver et al. [41], Robinson et al. [43] report that following the procedure of randomly assigned consent 25 % of the whole sample did not recall to have signed a consent form on participation in a genomic study. Furthermore, the majority (54 %) of research participants could not correctly report whom they had allowed to share the data with. In a similar direction point the data of Hoeyer et al. [37] who report that only 64.5 % of research participants in a Swedish biobank were aware of having consented to donate a blood sample, and 54 % did remember that they provided medical information by means of a questionnaire. Just 31.6 % knew that they could withdraw their consent. In the study of Watanabe and colleagues a third of participants (34.9 %) did not recall the contents of the consent procedure [45].

The study of Watanabe et al. [45] also reports findings about perceived understanding of the content of information provided as part of the consent procedure following three modes of conveying information about the research (DVD, brochure or personal discussion with researchers). Highest marks for understanding of the information about the biobank research were reported by participants after having personal discussion with the researchers. However, qualitative information reported back from the biobank researchers, who as part of the consent procedure informed the research participants, suggests that there is a perceived need to clarify information regarding the purpose of the research after they had given consent (“For what am I participating?”). The study of Ormond et al. [42] provides a detailed analysis with regards to understanding of information for different domains. By use of the “Quality of informed consent questionnaire (QuIC)” [52] and semi-structured questions the authors identify higher scores of understanding of research participants with regards to the nature of the study, benefit to future patients and the voluntary nature of participation. In contrast, research participants had lower knowledge scores with regards to potential risks and discomforts and the experimental nature of the research [42].

Cervo et al. [36] conducted the only intervention study in the sample of eligible articles for this review. The authors tested knowledge relevant to biobank research before and after a multisource informed consent procedure. The intervention consisted of an “enhanced consent form” and an information leaflet combined with two discussions between the potential biobank research participants and a physician and either a biobank-nurse or a biobank-biologists. While only 36.5 % knew what a biobank was before participating in the study, after this consent procedure knowledge about the purpose of the biobank and use of collected material was higher (>90 %).

### Expectations of benefits and perception of risks

Three of the eligible studies collected data on research participants’ expectations and perceptions regarding benefits and risks of the research and concerns related to biobanking. In the study by Ormond et al. [42] 10 % indicated having no expectation with regards to personally benefiting from research biobank’s findings, whereas Oliver et al. [41] report that 14.3 % ranked benefit of findings for research participants or family members as most important. In the study by Ormond et al. [42] 81 % agreed with the statement that the research was associated with no risk and discomfort. Seven per cent of the research participants were able to identify that learning potential genetic test results could be associated with risks [42]. Qualitative data analysed in this study, and also in the study of Oliver et al. [41] supports the quantitative findings on the difficulties of research participants to identify risks associated with the biobank research. In the qualitative study by Lipworth et al. [38] perceptions of trust and benefit dominated the narratives in comparison with the perception of informational risks.

## Discussion

This paper summarizes and analyzes data from socio-empirical studies on consent to biobank research. In comparison to a recently published systematic review [53], our paper focuses on the perceptions and preferences of research participants who underwent the consent procedure. Data on the views and preferences of participants in biobank research cannot be translated directly into ethical guidance [54]. However, experiences with the consent procedure and considerations made in this context can provide valuable insight which, in combination with normative analysis, can inform the debate about an ethically acceptable approach towards the practice of consent [55].

Our review suggests that there are two important issues related to how ethically acceptable consent can be elicited in research practice beyond the focus of the theoretical literature on justifications of broader or narrower approaches to consent. Firstly, the choices provided as part of the consent procedure, and secondly, the way in which potential research participants are informed about biobank research.

1. Offering a choice to research participants for an individualized consent
  From a series of qualitative and quantitative empirical research projects conducted by researchers of the Center for Medical Ethics and Health Policy, Baylor College of Medicine, Houston, Texas [39–41, 43] it could be shown that research participants are influenced in their decisions by the number of options presented as part of the consent procedure. All studies indicate that a considerable proportion of research participants chooses restricted or no data sharing after having learned that they can choose between a tiered form of consent compared to “traditional consent” (term used by the authors in this study) according to which the participants are requested to give broad consent or not to participate at all in the research. The fact that the three quantitative studies [40, 41, 43] were conducted with only one sample of research participants and focus on the question of consent for data sharing has to be taken into account as a limitation with regards to the representativeness of the findings. However, we argue that research participants should have a choice of scopes, for example, regarding the sharing of data. First of all, the confrontation with different consent options can be seen as one way to inform research participants about aspects which are less thought of by lay people, such as informational risks associated with biobank research. Secondly, such an approach acknowledges that individuals differ with regards to risk/benefit assessment relevant to decisions about participation in biobank research
2. Improving research participants’ understanding of risks
  The inability of research participants to recall information on biobank research and limited understanding with regard to the risks is a common feature of several studies included in this review. Limited recall or understanding is also a leitmotif of socio-empirical research on consent in the context of non-biobank research [56–59]. One particular challenge for research participants’ understanding may be that the benefits and risks in biobank research are asymmetrically distributed: Risks deriving from biobank research are of an individual and societal nature, whereas benefits are mainly for society as a whole, as biobank research does not usually produce any direct ready-made return for the donor. Potential risks are mainly represented by misuses of personal medical information resulting in possible discrimination and stigmatization. The knowledge of a person’s genetic makeup, for instance, can be used to justify unequal treatment: A candidate for a job may be excluded on the grounds of their genetic disposition to a future disease, or a person wishing to buy health insurance could be refused on genetic grounds [60]. Moreover, when participants in biobank research believe in a direct medical gain from the research activity itself, they incur a “therapeutic misconception” [61]. Therefore, the consent process for biobank research refers necessarily to an individual evaluation of individual risks and societal benefits [62]. The empirical data indicate that (non-existent) individual benefits are overestimated (i.e. therapeutic misconception), while many research participants are not aware that there are any risks at all (see, for example, Ormond et al. [42]. Given the abstract and complex nature of risks that could derive from biobank research, it seems ethically appropriate to offer such information to potential research participants in a way which allows them to decide whether they wish to receive more detailed information or not. A concrete measure in this respect would be to provide an electronic reminder, which summarizes information about the nature of biobank research to which they have consented, combined with an update on concrete research conducted or planned in the near future. This may provide research participants with an opportunity to learn more about what is done and to reconsider their initial decision(s) if they wish to do so. One of the models proposed to go beyond one-off static consent and to allow biobank participants to decide over time is dynamic consent, that is, a patient-centered approach with mechanisms of governance realized through information and communication technologies (ICT) solutions to allow participants to engage as much as they choose [63]. This approach should be contextualized within specific cultural and social settings (e.g. privacy laws could vary broadly between countries) and within specific networks and consortia [11]. Dynamic consent is a nuanced consent model, as it lets research participants go beyond the “all or nothing” option. Exemplarily, participants might control different privacy settings as flows of their personal information through a web interface, i.e. participants can decide who is allowed to access their de-identified information or who can have access to their contact information. Among the advantages of this approach, it is worth highlighting an improvement of public trust and an overcoming of the perennial issue of consent form length and comprehension—participants could, indeed, receive feedback on the general outcomes of the project. A dynamic consent approach can also improve transparency and accountability in the research processes: researchers can gather phenotypical information through continuous contact with patients that, in turn, by reducing research biases, can make the outcomes of research more effective and reliable [64]. Arguments against the dynamic consent approach are (i) its comparably greater management costs, which might not be feasible for less well funded groups or may escalate for very large research collaborations,, and (ii) its focus on ICT that, when exaggerated, could foster neither a dialogical interaction nor an enhancement of cognitive abilities of participants. In the wake of a dynamic consent approach, it would be ethically sound to address the wishes of biobank research participants, letting them deciding on issues that could influence their lives, for instance, allowing the control of fluxes of their personal information.
  As pointed out in the study of Watanabe et al. [45] and, in particular, in the pre-post study of Cervo et al. [36], the implementation of consent as a dialogical process supported by multisource information may be a measure to inform those patients who are interested more effectively. Given the huge technological input into biobank infrastructure, it is surprising how few innovative steps have been taken to test and evaluate measures to improve the consent procedures [25] and how little participants’ involvement has been developed to make biobank research sustainable [15]. According to the knowledge and practical experience of the authors, information about highly sophisticated biobank research is often provided in the form of a thick bundle of papers. Such research practice raises questions about setting priorities. Given the evidence on the impact of a more differentiated and enhanced approach towards information and consent [25], we argue that resources should not only be allocated to interdisciplinary research which take into account recent normative and empirical analysis on the issue of consent and biobank research, but also focus on the translation of these findings into an ethically acceptable research practice.

## Limitations

For reasons due to design of the study (i.e. selection of articles operated through a specific algorithm and through specific criteria as choosing only those studies where an informed consent was performed) and not least because a considerable amount of biobank research and accompanying ethico, legal and social studies are performed in Western countries, our results do not include socio-empirical data from the so-called ‘developing countries’ – the only article retrieved through our PubMed search on biobanking in developing countries has been excluded as not focused on informed consent [65]. This characteristic represents a limitation, as a review on socio-empirical literature about biobanking including data from developing countries would be of help to better understand implications for the conduct of consent in culture where there is, for example, an emphasis on oral communication and oral consent [66]. Furthermore, an analysis of biobanking in developing counties might be of help to foster policies around individual rights (i.e. the principle of individual autonomy), to activate mechanisms of citizenship consensus [67] and to encourage processes of community/public consultation [68]. In addition such research may be also relevant for the conduct of research in western countries where a more dialogical/oral approach communication could be considered as improvement to some of the currently used approaches. Moreover, this review of ten studies with differing sample size and methodology provides a limited insight into socio-empirical data on consent as perceived and viewed by research participants. More studies might have gained by using other databases and auxiliary search strategies. Due to limited resources and in light of the findings gained out of the retrieved studies we decided to limit our search strategy. Given that all studies stem from developed countries, and that 4 out of 10 studies are from a single center located in United States, where biobanking is a well-established practice, the findings of this review cannot be extrapolated to current biobank research in less developed or transitional countries or countries with different cultural values and a special attention should be paid for those countries with different traditions around informed consent for medical research.

## Conclusion

In recent years great parts of the discussion on ethical and legal aspects of consent for biobank research has focused on justifications for broader and narrower consent models [69]. The findings of this review suggest that, consent models which offer a more nuanced approach including choices of degrees to give or withhold consent to parts of biobank research reflect the preferences and views of research participants better than approaches which offer little choice regarding the scope of consent. In light of the lack of recall and limited understanding of information among research participants, research on the implementation and evaluation of improved consent procedures should be a priority. The rising use of information technologies in the context of evidence based decision support tools, as well as process oriented approaches towards information and consent are only two of several concrete examples which could serve the aim of informing potential biobank research participants appropriately about the issues at stake.

## References

[1] Hilgartner S, Gaudilliere JP, Rheinberger HJ (2004). Making maps and making social order. Governing American genome centers, 1988-93. From molecular genetics to genomics. The mapping cultures of twenty-century genetics.

[2] Mauron A, Elger B, Biller-Andorno N, Mauron A, Capron AM (2008). Biobanks, genomic and research. A nighmare for public policy makers?. Ethical issues in governing biobanks. Global perspectives.

[3] Scott CT, Caulfield T, Borgelt E, Illes J (2012). Personal medicine—the new banking crisis. Nat Biotechnol.

[4] Browman GP, Vollmann J, Virani A, Schildmann J (2014). Improving the quality of ‘personalized medicine’ research and practice: through an ethical lens. Pers Med.

[5] Dawson A (2003). Informed consent: should we really insist upon it?. New review of bioethics.

[6] Vollmann J, Winau R (1996). Informed consent in human experimentation before the Nuremberg code. BMJ (Clinical research ed).

[7] Sass HM (1983). Reichsrundschreiben 1931: pre-Nuremberg German regulations concerning new therapy and human experimentation. J Med Philos.

[8] Jonsen A (1998). The Birth of Bioethics.

[9] Caulfield T, Upshur RE, Daar A (2003). DNA databanks and consent: a suggested policy option involving an authorization model. BMC Med Ethics.

[10] Hofmann B, Solbakk JH, Holm S, Solbakk JH, Holm S, Hofmann B (2009). Consent to Biobank Research: One Size Fits All?. The Ethics of Research Biobanking.

[11] Kaye J, Whitley EA, Lund D, Morrison M, Teare H, Melham K (2014). Dynamic consent: a patient interface for twenty-first century research networks. Eur J Hum Genet.

[12] Stein DT, Terry SF (2013). Reforming biobank consent policy: a necessary move away from broad consent toward dynamic consent. Genet Test Mol Biomarkers.

[13] Casali PG (2014). Risks of the new EU Data Protection Regulation: an ESMO position paper endorsed by the European oncology community. Ann Oncol.

[14] European Commission. Background document: Public Consultation ‘Science 2.0’: Science in Transition. Directorates-General for Research and Innovation (RTD) and Communications Networks, content and Technology (CONNECT). 2014. http://ec.europa.eu/research/consultations/science-2.0/background.pdf. Accessed 05/09/2015.

[15] Simeon-Dubach D, Henderson MK (2014). Sustainability in biobanking. Biopreserv Biobank.

[16] Gaskell G, Gottweis H, Starkbaum J, Gerber MM, Broerse J, Gottweis U (2013). Publics and biobanks: Pan-European diversity and the challenge of responsible innovation. Eur J Hum Genet.

[17] Greely HT (1999). Breaking the stalemate: a prospective regulatory framework for unforseen research uses of human tissue samples and health information. Wake Forest Law Review.

[18] Hansson MG, Dillner J, Bartram CR, Carlson JA, Helgesson G (2006). Should donors be allowed to give broad consent to future biobank research?. Lancet Oncol.

[19] Helgesson G, Dillner J, Carlson J, Bartram CR, Hansson MG (2007). Ethical framework for previously collected biobank samples. Nat Biotechnol.

[20] Hansson MG, Levin M (2003). Biobanks as resources for health.

[21] Chadwick R, Berg K (2001). Solidarity and equity: new ethical frameworks for genetic databases. Nat Rev Genet.

[22] Hoedemaekers R, Gordijn B, Pijnenburg M (2007). Solidarity and justice as guiding principles in genomic research. Bioethics.

[23] Knoppers BM, Chadwick R (2005). Human genetic research: emerging trends in ethics. Nat Rev Genet.

[24] Petrini C (2010). “Broad” consent, exceptions to consent and the question of using biological samples for research purposes different from the initial collection purpose. Soc Sci Med.

[25] Kohnen T, Schildmann J, Vollmann J, Braun M, Dabrock P (2013). Patients’ self-determination in “personalised medicine”: The case of whole genome sequencing and tissue banking in oncology. Individualised Medicine “between hype und hope”.

[26] Beauchamp TL (2011). Informed consent: its history, meaning, and present challenges. Camb Q Healthc Ethics.

[27] Schildmann E, Schildmann J (2014). Palliative sedation therapy: a systematic literature review and critical appraisal of available guidance on indication and decision making. J Palliat Med.

[28] Schildmann J. Decisions about limiting treatment in cancer patients. A systematic review and clinical-ethical analysis of reported variables. J Palliat Med. 2015. doi:10.1089/jpm.2014.0441. 10.1089/jpm.2014.0441 26248019

[29] Schildmann J, Vollmann J, Gordon PDJS, Vollmann PJ, Schildmann J (2013). Evaluation of clinical ethics consultation: a systematic review and critical appraisal of research methods and outcome criteria. Clinical Ethics Consultation: Theories and Methods.

[30] Boniolo G, Di Fiore PP, Pece S (2012). Trusted consent and research biobanks: towards a ‘new alliance’ between researchers and donors. Bioethics.

[31] Cambon-Thomsen A, Rial-Sebbag E, Knoppers BM (2007). Trends in ethical and legal frameworks for the use of human biobanks. Eur Respir J.

[32] Hofmann B (2009). Broadening consent—and diluting ethics?. J Med Ethics.

[33] Mascalzoni D, Hicks A, Pramstaller P, Wjst M (2008). Informed consent in the genomics era. PLoS Med.

[34] Sheehan M (2011). Can broad consent be informed consent?. Public Health Ethics.

[35] Philipp M (2000). Qualitative inhaltsanalyse. Forum Qualitative Sozialforschung/Forum: Qualitative Social Research.

[36] Cervo S, Rovina J, Talamini R, Perin T, Canzonieri V, De Paoli P (2013). An effective multisource informed consent procedure for research and clinical practice: an observational study of patient understanding and awareness of their roles as research stakeholders in a cancer biobank. BMC Med Ethics.

[37] Hoeyer K, Olofsson BO, Mjorndal T, Lynoe N (2005). The ethics of research using biobanks—reason to question the importance attributed to informed consent. Arch Intern Med.

[38] Lipworth W, Morrell B, Irvine R, Kerridge I (2009). An empirical reappraisal of public trust in biobanking research: rethinking restrictive consent requirements. J Law Med.

[39] McGuire AL, Hamilton JA, Lunstroth R, McCullough LB, Goldman A (2008). DNA data sharing: research participants’ perspectives. Genet Med.

[40] McGuire AL, Oliver JM, Slashinski MJ, Graves JL, Wang T, Kelly PA (2011). To share or not to share: a randomized trial of consent for data sharing in genome research. Genet Med.

[41] Oliver JM, Slashinski MJ, Wang T, Kelly PA, Hilsenbeck SG, McGuire AL (2012). Balancing the risks and benefits of genomic data sharing: genome research participants’ perspectives. Public Health Genomics.

[42] Ormond KE, Cirino AL, Helenowski IB, Chisholm RL, Wolf WA (2009). Assessing the understanding of biobank participants. Am J Med Genet A.

[43] Robinson JO, Slashinski MJ, Wang T, Hilsenbeck SG, McGuire AL (2013). Participants’ recall and understanding of genomic research and large-scale data sharing. J Empir Res Hum Res Ethics.

[44] Valle-Mansilla JI, Ruiz-Canela M, Sulmasy DP (2010). Patients’ attitudes to informed consent for genomic research with donated samples. Cancer Invest.

[45] Watanabe M, Inoue Y, Chang C, Hong H, Kobayashi I, Suzuki S (2011). For what am I participating? The need for communication after receiving consent from biobanking project participants: experience in Japan. J Hum Genet.

[46] Hobbs A, Starkbaum J, Gottweis U, Wichmann HE, Gottweis H (2012). The privacy-reciprocity connection in biobanking: comparing German with UK strategies. Public Health Genomics.

[47] Ridgeway JL, Han LC, Olson JE, Lackore KA, Koenig BA, Beebe TJ (2013). Potential bias in the bank: what distinguishes refusers, nonresponders and participants in a clinic-based biobank?. Public Health Genomics.

[48] Steinsbekk KS, Ursin LO, Skolbekken JA, Solberg B (2013). We’re not in it for the money-lay people’s moral intuitions on commercial use of ‘their’ biobank. Med Health Care Philos.

[49] Williams PH, Nemeth LS, Sanner JE, Frazier LQ (2013). Thematic analysis of cardiac care patients’ explanations for declining contribution to a genomic research-based biobank. Am J Crit Care.

[50] Rahm AK, Wrenn M, Carroll NM, Feigelson HS (2013). Biobanking for research: a survey of patient population attitudes and understanding. J Community Genet.

[51] Tindana P, Bull S, Amenga-Etego L, de Vries J, Aborigo R, Koram K (2012). Seeking consent to genetic and genomic research in a rural Ghanaian setting: a qualitative study of the MalariaGEN experience. BMC Med Ethics.

[52] Joffe S, Cook EF, Cleary PD, Clark JW, Weeks JC (2001). Quality of informed consent: a new measure of understanding among research subjects. J Natl Cancer Inst.

[53] Khan A, Capps BJ, Sum MY, Kuswanto CN, Sim K (2014). Informed consent for human genetic and genomic studies: a systematic review. Clin Genet.

[54] Salloch S, Vollmann J, Schildmann J (2014). Ethics by opinion poll? The functions of attitudes research for normative deliberations in medical ethics. J Med Ethics.

[55] Salloch S, Schildmann J, Vollmann J (2012). Empirical research in medical ethics: how conceptual accounts on normative-empirical collaboration may improve research practice. BMC Med Ethics.

[56] Bergler JH, Pennington AC, Metcalfe M, Freis ED (1980). Informed consent: how much does the patient understand?. Clin Pharmacol Ther.

[57] Cassileth BR, Zupkis RV, Sutton-Smith K, March V (1980). Informed consent—why are its goals imperfectly realized?. N Engl J Med.

[58] Estey A, Wilkin G, Dossetor J (1994). Are research subjects able to retain the information they are given during the consent process?. Health Law Review.

[59] Hassar M, Weintraub M (1976). “Uniformed” consent and the wealthy volunteer: an analysis of patient volunteers in a clinical trial of a new anti-inflammatory drug. Clin Pharmacol Ther.

[60] Ethikrat N (2010). Human biobanks for research.

[61] Henderson GE, Churchill LR, Davis AM, Easter MM, Grady C, Joffe S (2007). Clinical trials and medical care: defining the therapeutic misconception. PLoS Med.

[62] Hoeyer K, Tutton R, Corrigan O (2004). Ambiguous gifts. Public anxiety, informed consent and biobanks. Genetic databases. Socio-ethical issues in the collection and use of DNA.

[63] Terry SF, Shelton R, Biggers G, Baker D, Edwards K (2013). The haystack is made of needles. Genet Test Mol Biomarkers.

[64] Li AM, Terry SF (2015). Linking personal health data to genomic research. Genet Test Mol Biomarkers.

[65] Barr M, Souan L, MacGabhann P, Muller J, Al Ashhab M, Jasser M (2014). The establishment of an ISO compliant cancer biobank for Jordan and its neighboring countries through knowledge transfer and training. Biopreserv Biobank.

[66] Onvomaha Tindana P, Kass N, Akweongo P (2006). The informed consent process in a rural African setting: a case study of the Kassena-Nankana district of Northern Ghana. IRB.

[67] Ahram M, Othman A, Shahrouri M (2012). Public support and consent preference for biomedical research and biobanking in Jordan. Eur J Hum Genet.

[68] Rotimi C, Leppert M, Matsuda I, Zeng C, Zhang H, Adebamowo C (2007). Community engagement and informed consent in the International HapMap project. Community Genet.

[69] D'Abramo F. Biobank research, informed consent and society. Towards a new alliance? J Epidemiol Community Health. 2015. doi:10.1136/jech-2014-205215. 10.1136/jech-2014-205215 25669218

